# Fish oil and krill oil differentially modify the liver and brain lipidome when fed to mice

**DOI:** 10.1186/s12944-015-0086-2

**Published:** 2015-08-11

**Authors:** Jon Skorve, Mika Hilvo, Terhi Vihervaara, Lena Burri, Pavol Bohov, Veronika Tillander, Bodil Bjørndal, Matti Suoniemi, Reijo Laaksonen, Kim Ekroos, Rolf K. Berge, Stefan E. H. Alexson

**Affiliations:** Department of Clinical Science, University of Bergen, N-5021 Bergen, Norway; Department of Heart Disease, Haukeland University Hospital, N-5021 Bergen, Norway; Division of Clinical Chemistry, Department of Laboratory Medicine, Karolinska Institutet, Karolinska University Hospital, S-14186 Stockholm, Sweden; Zora Biosciences Oy, Biologinkuja 1, 02150 Espoo, Finland; Present address: Aker BioMarine ASA, Fjordalléen 16, NO-0115 Oslo, Norway

**Keywords:** Lipidomic analysis, Krill and fish oil, Omega-3 fatty acids, Sphingolipids, Fatty acid composition

## Abstract

**Background:**

Marine food is an important source of omega-3 fatty acids with beneficial health effects. Oils from marine organisms have different fatty acid composition and differ in their molecular composition. Fish oil (FO) has a high content of eicosapentaenoic and docosahexaenoic acids mainly esterified to triacylglycerols, while in krill oil (KO) these fatty acids are mainly esterified to phospholipids. The aim was to study the effects of these oils on the lipid content and fatty acid distribution in the various lipid classes in liver and brain of mice.

**Methods:**

Mice were fed either a high-fat diet (HF), a HF diet supplemented with FO or with KO (n = 6). After six weeks of feeding, liver and brain lipid extracts were analysed using a shotgun and TAG lipidomics approach. Student *t*-test was performed after log-transformation to compare differences between study groups.

**Results:**

Six weeks of feeding resulted in significant changes in the relative abundance of many lipid classes compared to control mice. In both FO and KO fed mice, the triacylglycerol content in the liver was more than doubled. The fatty acid distribution was affected by the oils in both liver and brain with a decrease in the abundance of 18:2 and 20:4, and an increase in 20:5 and 22:6 in both study groups. 18:2 decreased in all lipid classes in the FO group but with only minor changes in the KO group. Differences between the feeding groups were particularly evident in some of the minor lipid classes that are associated with inflammation and insulin resistance. Ceramides and diacylglycerols were decreased and cholesteryl esters increased in the liver of the KO group, while plasmalogens were decreased in the FO group. In the brain, diacylglycerols were decreased, more by KO than FO, while ceramides and lactosylceramides were increased, more by FO than KO.

**Conclusion:**

The changes in the hepatic sphingolipids and 20:4 fatty acid levels were greater in the KO compared to the FO fed mice, and are consistent with a hypothesis that krill oil will have a stronger anti-inflammatory action and enhances insulin sensitivity more potently than fish oil.

## Background

Marine omega-3 fatty acids are well known for a range of documented health benefits [1]. Omega-3 polyunsaturated fatty acids (PUFAs) such as eiocosapentaenoic acid (EPA) and docosahexaenoic acid (DHA) are bioactive dietary compounds that are found particularly in marine-derived food sources such as fatty fish, seaweed, shellfish, microalgae and krill. Marine oils differ in their fatty acid composition and lipid structure, although EPA and DHA are the predominant fatty acids. Most fish oils (FOs) on the market today have their omega-3 PUFAs incorporated into triacylglycerols (TAGs) or in ethyl esters. Krill oil (KO), extracted from Antarctic krill (Euphausia superba), has a unique chemical composition and contains astaxanthin, which due to its anti-oxidative effect, might enhance the stability of the omega-3 PUFAs in the oil and thereby preserve them from lipid oxidation [2, 3]. Like FO, it is rich in omega-3 PUFAs, but the fatty acids (FAs) are mainly incorporated into phospholipids (PLs) rather than TAGs, with some content of un-esterified fatty acids. This may be biologically and therapeutically significant, since PL FAs are well-absorbed by the intestine and readily incorporated into cell membranes [4–7]. These fatty acids, whether being presented as a part of TAG molecules (like in FO) or incorporated into PL molecules (as in KO), are capable of influencing different metabolic pathways [8] and differently modulate physiological effects [9]. However, also specific PLs may have distinct properties in relation to bioavailability, intestinal absorption and stability, which may be of importance in relation to benefits for metabolic health [10]. The structural differences in the PUFA-rich lipid molecules may affect tissue uptake and the distribution of fatty acids in cellular lipid fractions and thereby promote different regulatory effects on lipid homeostasis [11, 12].

We have previously conducted a study to investigate the metabolic effects in mice when two of the major sources of omega-3 supplements, FO and KO, were supplemented to a Western-like high-fat diet. Equal amounts of FO and KO (6 % by weight) were added to the diet, and the effects on plasma and liver lipids as well as gene regulation in liver and intestine were investigated [13]. In spite of a lower omega-3 fatty acid content in the KO supplemented diet, plasma and liver PLs omega-3 levels were similar in the two groups, indicating a higher bioavailability of omega-3 fatty acids from KO. Moreover, feed containing either FO or KO promoted different gene expression profiles in liver and intestine with FO causing an apparent PPARα response, while KO supplementation rather acted as a negative regulator of endogenous cholesterol and fatty acid synthesis. It was concluded from this study that both FO and KO promote lowering of plasma lipids and regulate lipid homeostasis, but with different efficacy and partially *via* different mechanisms. To further explore the mechanistic basis for these differences between the two oils, we have here conducted a comprehensive lipidomic analysis of livers from these mice. Omega-3 fatty acids as well as astaxanthin are stated to have important functions also in the brain as neuroprotectors during inflammation and oxidative stress [for reviews, see refs 14, 15]. We therefore analyzed the effects of omega-3 PUFA’s provided as FO and KO on the fatty acid composition also in brain lipids with special focus on DHA, which is considered to have important functions in the brain. This analysis showed that feeding mice with either FO or KO differentially affected the lipid and fatty acid composition in liver and brain.

## Results

### Body weight and feed intake

The amount of FO and KO in the diets were approximately the same. However, the FO contained more omega-3 FAs than the KO with EPA and DHA levels approximately twice as high as in the KO diet (Table 1). The basic outcomes such as food intake, body weights and plasma lipids were recently reported [13]. The final weights of the mice were not significantly different between the three study groups. Also the feed intake in the two marine oil diet groups was similar in comparison with the control group.

**Table 1:** Fat content and fatty acid composition of the diets

	High fat control	Fish Oil (FO)	Krill Oil (KO)
Fat source (% in diet)			
Lard	21,3 %	15,7 %	15,6 %
Soy oil	2,3 %	2,3 %	2,3 %
Fish/krill oil		5,8 %	5,6 %
Fatty acids (% of total fatty acids in diet)			
Total SFA	42,9 %	34,1 %	39,7 %
Total MUFA	38,7 %	32,1 %	35,4 %
Total ω6	16,4 %	14,5 %	14,6 %
EPA	0,03 %	9,0 %	5,2 %
DHA	0,05 %	6,4 %	2,3 %

The content of EPA and DHA in the KO diet was about half of the FO diet

### Distribution of lipid classes in liver and brain

**Fig. 1:**
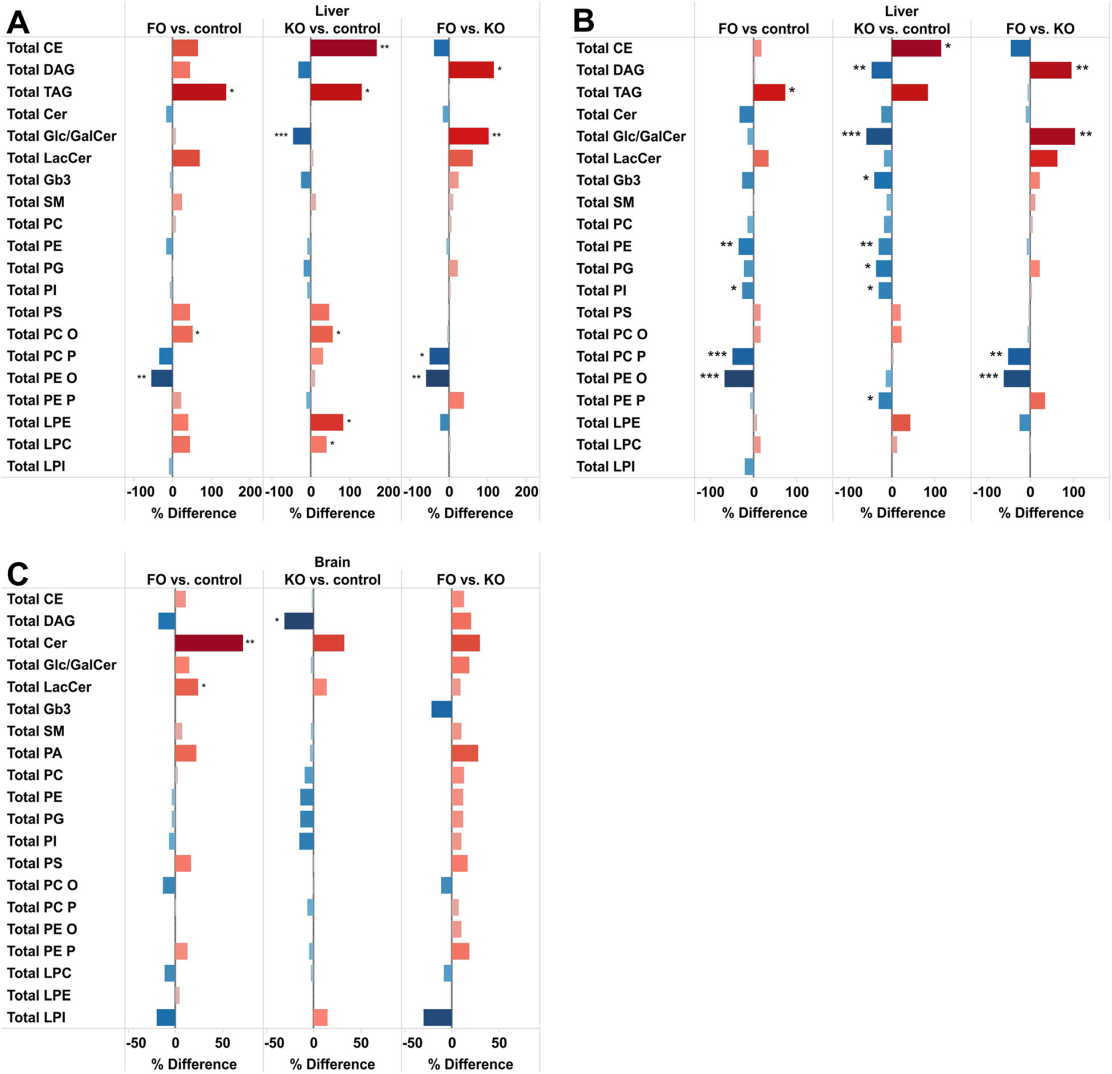
Relative differences in liver and brain lipid classes. Differences between FO, KO and control mice based on **a** concentration in liver, **b** mole percentage in liver and **c** concentration in brain. *** p < 0.001, ** p < 0.01, * p < 0.05

After 6 weeks, relative hepatic TAG content showed a significant nearly 2-fold increase in both FO and KO fed animals as compared to control mice (Table 2 and Fig. 1). This increase affected the relative distribution of the lipid classes, and therefore, the distribution of the lipid classes are reported both as concentration and mole percentages for liver (Table 2) and brain (Table 3).

**Table 2:** Concentration and mole percentage of lipid classes in liver

	Concentration (pmol/ug tissue)						Mol %					
	Control		Fish Oil (FO)		Krill Oil (KO)		Control		Fish Oil (FO)		Krill Oil (KO)	
Lipid class	Average	SD	Average	SD	Average	SD	Average	SD	Average	SD	Average	SD
CE	0,287	0,116	0,477	0,297	0,776	0,371	1,8	0,7	2,3	1,1	4,1	2,3
DAG	0,495	0,163	0,714	0,399	0,330	0,067	3,1	0,8	3,4	1,1	1,7	0,3
PC	7,454	2,215	8,097	1,926	7,550	1,762	46,2	5,8	41,1	4,1	39,2	9,3
PE	1,853	0,421	1,546	0,500	1,669	0,255	11,8	1,5	7,9	1,7	8,7	1,2
PG	0,039	0,011	0,039	0,011	0,032	0,011	0,2	0,0	0,2	0,0	0,2	0,1
PI	0,115	0,023	0,108	0,029	0,103	0,034	0,7	0,1	0,6	0,1	0,5	0,2
PS	0,780	0,379	1,129	0,298	1,158	0,333	4,8	1,5	5,8	1,1	6,0	1,6
PC O	0,238	0,054	0,360	0,093	0,374	0,114	1,5	0,2	1,8	0,2	1,9	0,6
PC P	0,059	0,018	0,039	0,012	0,077	0,024	0,4	0,1	0,2	0,1	0,4	0,1
PE O	0,018	0,005	0,008	0,003	0,020	0,005	0,1	0,0	0,0	0,0	0,1	0,0
PE P	0,029	0,006	0,035	0,014	0,025	0,012	0,2	0,0	0,2	0,0	0,1	0,1
LPC	0,110	0,027	0,161	0,038	0,156	0,022	0,7	0,1	0,8	0,2	0,8	0,1
LPE	0,014	0,005	0,019	0,007	0,025	0,008	0,1	0,0	0,1	0,0	0,1	0,0
LPI	0,003	0,001	0,003	0,001	-	-	-	-	-	-	-	-
SM	0,216	0,049	0,271	0,085	0,245	0,075	1,4	0,2	1,4	0,4	1,3	0,3
Cer	1,439	0,275	1,208	0,276	1,431	0,238	9,4	2,3	6,5	2,8	7,4	0,9
Glc/GalCer	0,613	0,125	0,673	0,176	0,332	0,051	4,0	1,2	3,5	1,3	1,8	0,4
LacCer	0,013	0,004	0,023	0,011	0,014	0,004	0,1	0,0	0,1	0,1	0,1	0,0
Gb3	0,015	0,004	0,014	0,004	0,011	0,003	0,1	0,0	0,1	0,0	0,1	0,0
TAG	2,158	1,109	5,145	3,012	5,021	2,725	13,3	5,4	24,0	8,7	25,6	12,9

**Table 3:** Concentration and mole percentage of total lipid classes in brain

	Concentration (pmol/ug tissue)						Mol %					
	Control		Fish Oil (FO)		Krill Oil (KO)		Control		Fish Oil (FO)		Krill Oil (KO)	
Lipid class	Average	SD	Average	SD	Average	SD	Average	SD	Average	SD	Average	SD
CE	0,049	0,006	0,055	0,010	0,048	0,006	0,2	0,0	0,2	0,0	0,2	0,0
DAG	0,149	0,040	0,122	0,030	0,102	0,017	0,5	0,1	0,4	0,1	0,4	0,1
PC	18,503	1,473	18,891	1,964	16,771	0,890	60,5	2,0	58,9	4,2	59,3	2,1
PE	2,262	0,204	2,169	0,447	1,942	0,277	7,4	0,8	6,7	1,0	6,9	1,0
PG	0,082	0,012	0,079	0,024	0,071	0,019	0,3	0,0	0,2	0,1	0,3	0,1
PI	0,196	0,032	0,183	0,031	0,166	0,026	0,6	0,1	0,6	0,1	0,6	0,1
PS	4,923	0,649	5,754	1,063	4,935	0,525	16,1	1,1	17,8	2,1	17,4	1,5
PC O	0,073	0,035	0,064	0,011	0,073	0,022	0,2	0,1	0,2	0,0	0,3	0,1
PC P	0,089	0,013	0,089	0,012	0,083	0,014	0,3	0,0	0,3	0,0	0,3	0,0
PE O	-	-	0,012	0,002	0,011	0,002	-	-	0,0	0,0	0,0	0,0
PE P	1,957	0,464	2,199	0,459	1,855	0,194	6,3	1,1	6,8	1,1	6,6	0,8
LPC	0,032	0,008	0,028	0,006	0,031	0,005	0,1	0,0	0,1	0,0	0,1	0,0
LPE	0,011	0,005	0,012	0,003	-	-	-	-	-	-	-	-
LPG	0,048	0,024	-	-	-	-	0,2	0,1	-	-	-	-
LPI	0,025	0,013	0,020	0,007	0,029	0,007	0,1	0,0	0,1	0,0	0,1	0,0
SM	1,448	0,141	1,545	0,169	1,400	0,171	4,8	0,7	4,8	0,2	4,9	0,6
Cer	0,080	0,029	0,136	0,020	0,105	0,026	0,3	0,1	0,4	0,0	0,4	0,1
Glc/GalCer	0,571	0,114	0,655	0,102	0,555	0,107	1,9	0,3	2,1	0,4	2,0	0,4
LacCer	0,022	0,002	0,027	0,004	0,025	0,005	0,1	0,0	0,1	0,0	0,1	0,0

PLs constitute the main lipid class in the liver, with phosphatidylcholines (PCs) and phosphatidylethanolamines (PEs) being the main PL species (Table 2). PC (concentration or mole %) did not show any difference between the groups, but total PE and phosphatidylinositol (PI) concentrations were slightly decreased in both FO and KO groups (Fig. 1a). However, the relative abundance of PE and PI were both significantly decreased in both diet groups due to the increases in TAGs (Fig. 1b). In general the differences between the diet groups were small, however, the ether phospholipids PC P and PE O were both decreased in the FO mice, while slightly increased in the KO mice with the absolute and relative concentrations being significantly lower in the FO group compared to KO (Fig. 1a, b). Also, the concentrations of LPE and LPC were significantly increased in KO mice but not in FO mice.

Cholesteryl esters were more than doubled in KO supplemented mice but only slightly increased in FO mice (Fig. 1a, b). Also for the diacylglyerol (DAG) lipids there was a distinct difference between the two study groups with the relative abundance of total DAGs being decreased nearly 50 % in the KO group but slightly increased in the FO group with the relative concentration being significantly different between FO and KO (Fig. 1a, b).

All the ceramides, except lactosylceramide (LacCer) showed a decreasing trend in their relative abundance in both FO and KO fed mice. For glucosyl/galactosylceramides the decrease was particularly large in the KO mice (nearly 60 %), and also globotriaosylceramides (Gb3s) showed a statistically significant decrease. The abundance of LacCer changed in opposite directions with a 40 % increase in the FO group and 20 % decrease in the KO group, although the differences were not significant (Fig. 1b).

Brain contains about 25 % of the body’s cholesterol of which almost all is unesterified. Here we only analyzed esterified cholesterol, which was essentially unchanged in both oil groups. The dominating lipid class was PCs, constituting approximately 60 mole percent of total lipids, while PSs constituted approximately 16–18 mole percent (Table 3). TAGs were not analyzed as their abundance in brain is very low, however DAG was significantly decreased in KO (Fig. 1c). There were only small changes in the levels of the major lipid classes, but total Cer and LacCer, which form minor lipid components in the brain, were increased in FO (Fig. 1c). There was a trend (but not significantly different) towards opposite regulation in e.g., lysophosphatidylinositol (LPI), phosphatidic acid (PA) and PE P.

### Distribution of fatty acids and lipids in liver

**Fig. 2:**
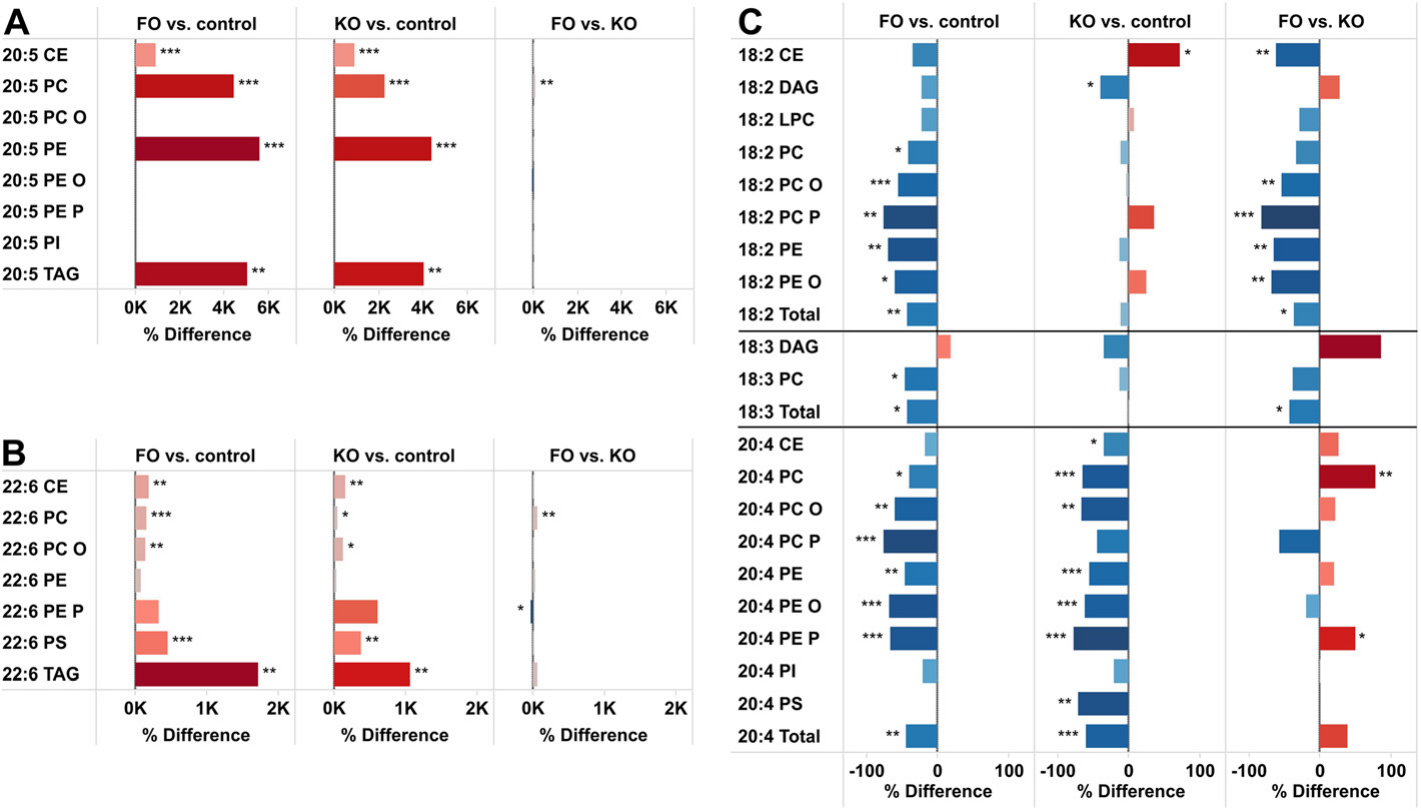
Relative differences in liver concentrations of polyunsaturated fatty acids. Differences in liver lipid classes between FO, KO and control mice for the following fatty acids 20:5 (**a**), 22:6, (**b**), 18:2, 18:3 and 20:4 (**c**). *** p < 0.001, ** p < 0.01, * p < 0.05

The lipidomic approach identifies lipid species, however, due to the analytical challenge of identification of molecular TAG species, TAG lipids are reported as the sum of C-atoms and double bonds (i.e., TAG 52:3 contains a mixture of TAG (16:0/18:1/18:2) and TAG (16:1/18:1/18:1)). Figure 2 summarizes the changes in fatty acid composition of some of the most common lipid species in liver. As expected, the relative content of the polyunsaturated n-3 fatty acids EPA (20:5) and DHA (22:6) was strongly increased in e.g., PC, TAG, PE and CE (Fig. 2a, b). The increases were largest in PC, PE and TAG for EPA (about 50-fold) with the effect being strongest with FO. The effects of FO and KO were very similar with the difference between the FO and KO being rather small compared to the overall changes caused by marine oil feeding. DHA was increased in several lipid classes, i.e., CE, PC, PC O, PS and TAG, although the increase in DHA was less evident compared to EPA (Fig. 2b). The increased concentrations of EPA and DHA in the various lipid classes were mainly at the expense of 18:2, 18:3 and 20:4 (Fig. 2c). The total levels of 18:2, 18:3 and 20:4 were decreased by FO whereas only total 20:4 was decreased by KO. In PC, PE,

PC O, PC P and PE O the decrease in 18:2 was significant in the FO group. In the KO mice the changes were minor with 18:2 being increased in CE and decreased in DAG only. Total 18:3 and 18:3 in PC was decreased by FO while 20:4 was decreased in most PL classes in both groups with the effect being stronger with KO.

**Fig. 3:**
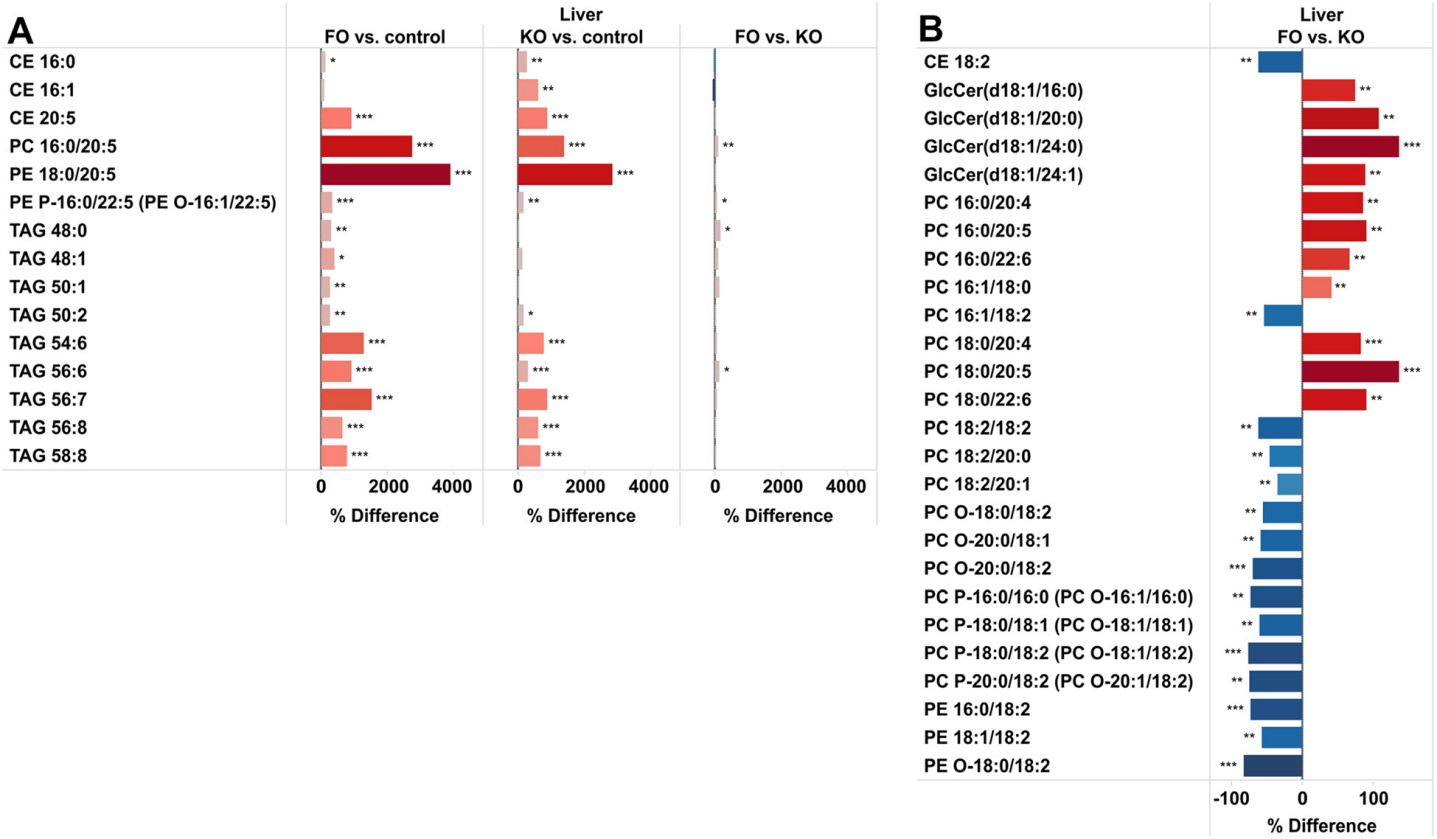
Relative differences in liver lipids which show a statistically significant change. **a** Lipids with a statistically significant relative difference > 250 in either FO or KO group as compared to the control group. **b** Lipids that had p-value < 0.01 between the FO and KO groups. *** p < 0.001, ** p < 0.01, * p < 0.05

**Fig. 4:**
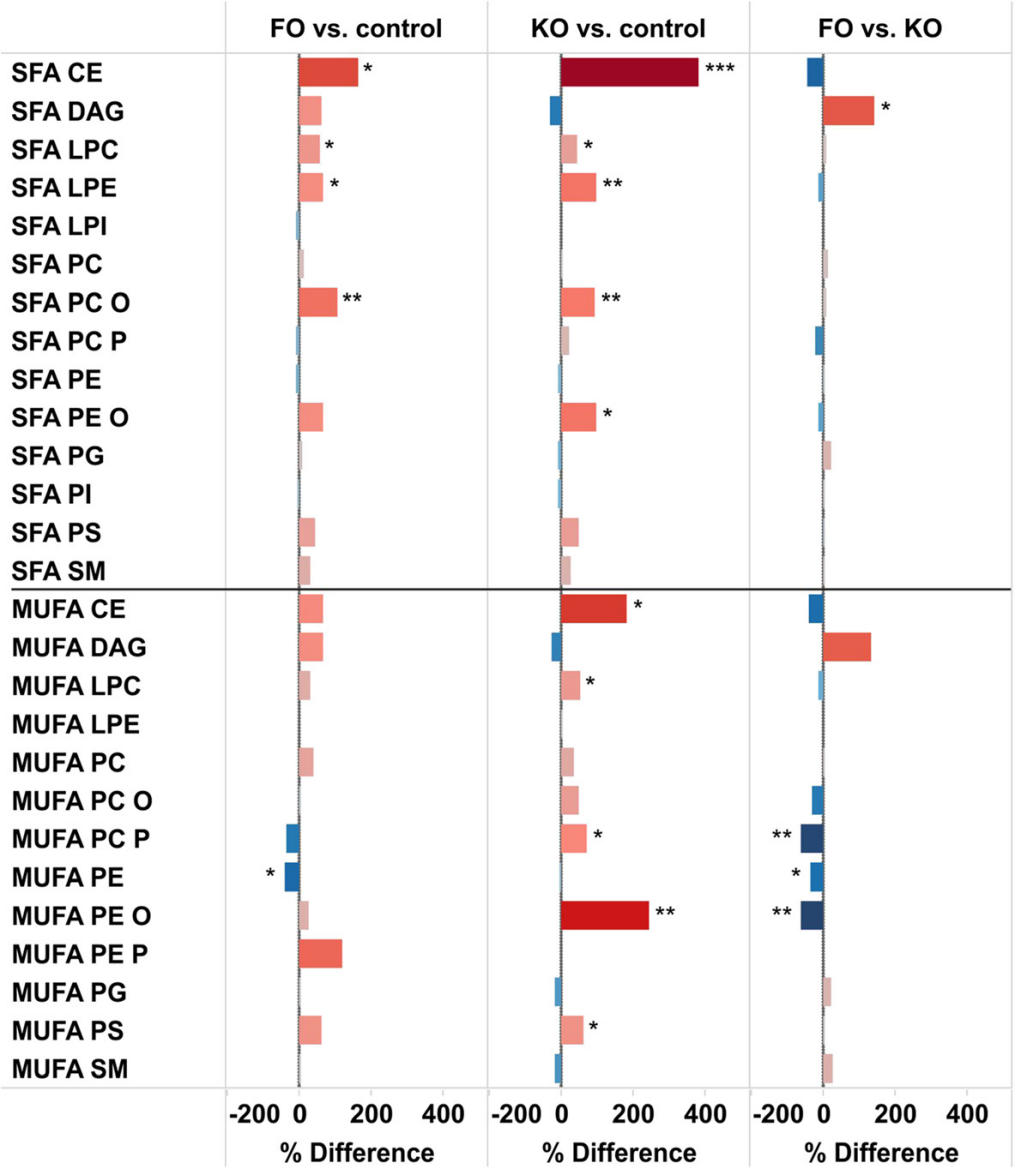
Relative differences in liver concentrations of saturated (SFA) and monounsaturated (MUFA) fatty acids. Differences in liver lipid classes between FO, KO and control mice. *** p < 0.001, ** p < 0.01, * p < 0.05

As shown in Fig. 1a, the total level of CE was increased more in KO than in FO mice, while CE 20:5 and 22:6 were increased to similar extent in both groups (Fig. 2a, b). The increased level of CE in KO is mainly due to larger increases in SFA and MUFA CEs, such as CE 16:0, CE 16:1 (Figs. 3a and 4). At the molecular level, the most significantly affected lipids were PC 16:0/20:5 and PE 18:0/20:5 (Fig. 3a). TAGs containing SFAs and MUFAs were increased only in the FO group while PUFA-containing TAGs were increased in both groups (Fig. 3a). Figure 3b summarizes the most strongly (p < 0.01) regulated lipids between FO and KO. Several 18:2 containing lipids and ether phospholipids were lower in the FO group, whereas glucosylceramides (GlcCer) were increased in the FO group as compared to the KO group.

Figure 4 summarizes the changes in lipids containing SFAs and MUFAs. There was an increase in the abundance of SFA containing LPC, LPE, PC O, PE O and PS, with small differences between the study groups. For MUFA-containing lipids, statistically significant increases were observed in LPC, PC P, PE O and PS in the KO group, while a decrease in PE was observed in the FO group.

### Distribution of fatty acids and lipids in brain

**Fig. 5:**
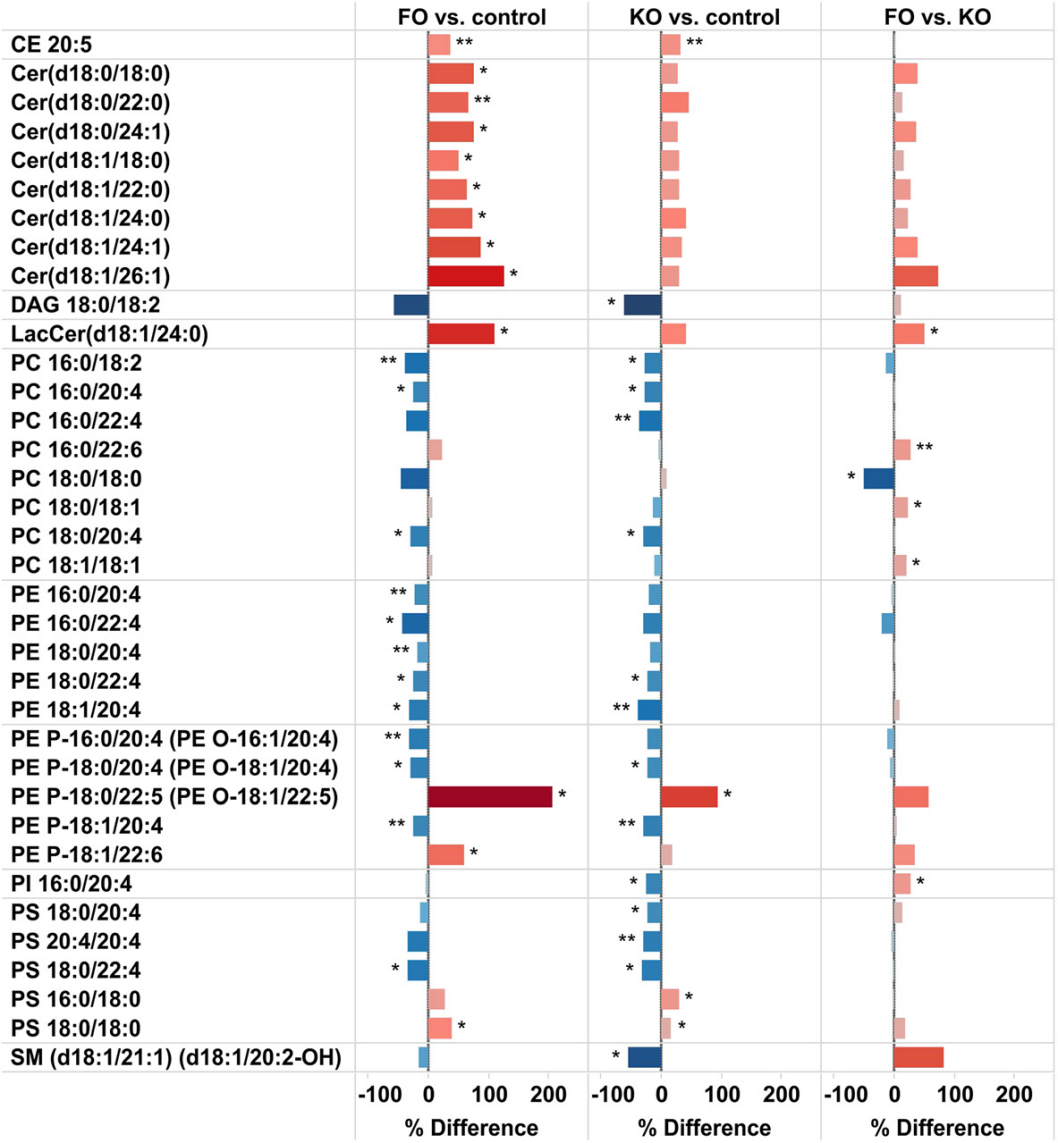
Relative differences in concentrations of brain lipids. Lipids that showed statistically significanct changes in any of the comparisons between FO, KO and control mice are shown. ** p < 0.01, * p < 0.05

**Fig. 6:**
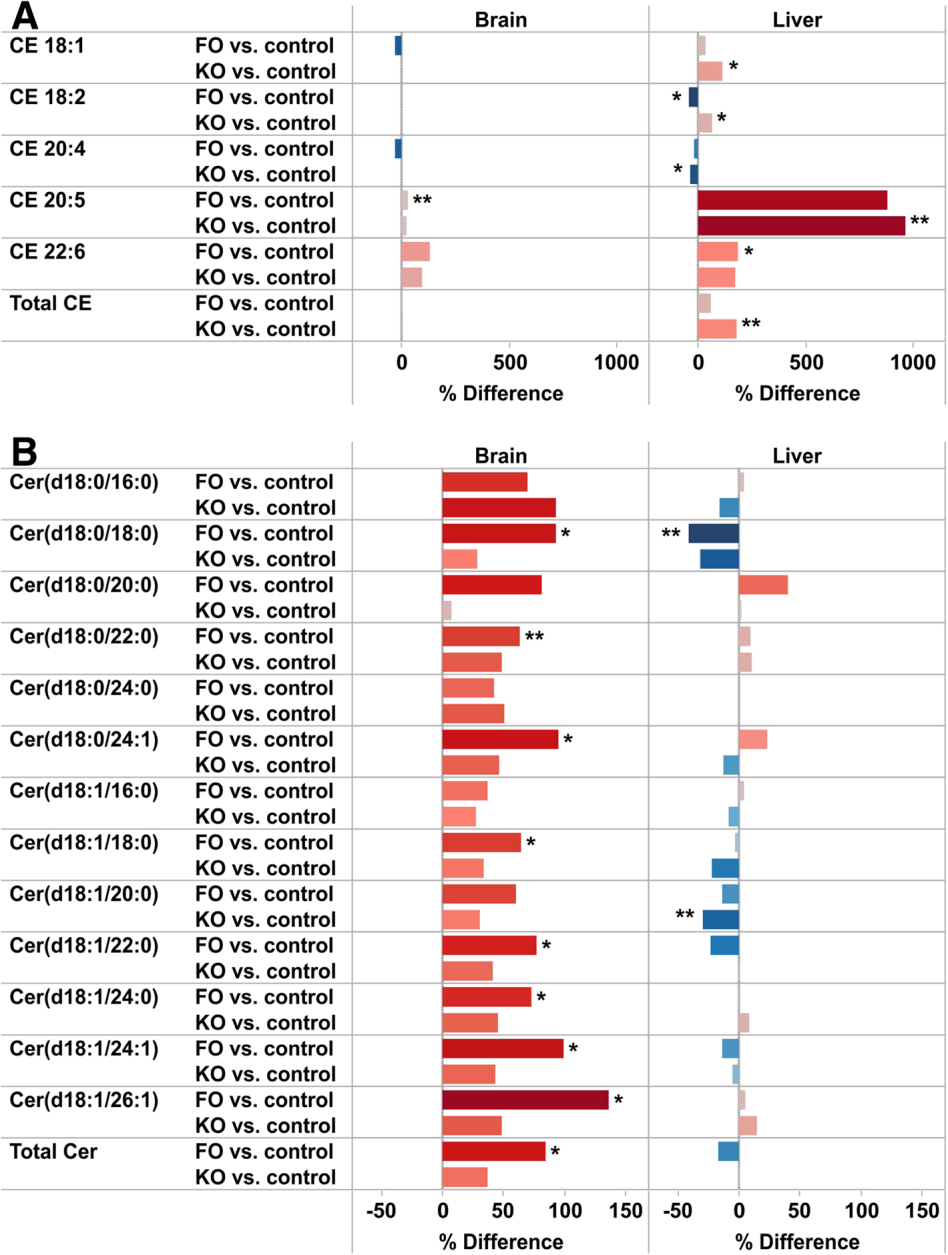
Comparison of relative differences in liver and brain lipids. Differences in **a** cholesteryl esters and **b** ceramides between FO, KO and control mice. ** p < 0.01, * p < 0.05

Significant changes could be observed for specific lipids in brain, although the changes in the abundance of the major lipid classes were small. Figure 5 summarizes the changes that were significantly different by FO or KO. CE 20:5 increased by both FO and KO, and, surprisingly, the concentration of CE 20:5 (which is the main CE in brain) was about 20 times higher than CE 22:6 in brain in the HF fed mice. Both CEs were increased by FO and KO, and although CE 22:6 increased more (about 2-fold) than CE 20:5 (about 20 %) (Fig. 6a), 20:5 remained the main CE species in brain. FO decreased CE 18:1 and 20:4 and of interest to note is that only 20:5 and 22:6 CEs were detected in KO fed mice, the other CEs being below the detection level. The fatty acid profile as well as regulation of CE in liver and brain is markedly different with e.g., CE 18:1 and 18:2 being increased by KO in liver (Fig. 6a). Several of the Cer fatty acid species in brain increased significantly by FO, while the increases in KO were weaker and not significant (Fig. 5). Several PLs (PC, PE, PE P/PE O and PS), in particular containing 20:4, were decreased in both groups with the exception of 22:5 and 22:6 containing PE P species that were increased in particular in the FO group. DAG 18:0/18:2, PC16:0/22:4, PI 16:0/20:4, PS 18:0/20:4, PS 20:4/20:4 and SM (d18:1/21:1) (d18:1/20:2-OH) decreased in both groups, but significantly only in KO group (Fig. 5). 20:5 was not detected in PL in the control mice, but trace amounts were detected in the FO and KO mice, whereas 22:6 was much more abundant in several PL. 20:5 was not detected in any PE P species, but 22:5 and 22:6 were increased by FO and KO in some ether-linked PE Ps (PE P-18:0/22:5 (PE O-18:1/22:5) and PE P-18:1/22:6, Fig. 5).

## Discussion

Fish oil and krill oil are both abundant in omega-3 fatty acids but the relative content of the main fatty acid species and the molecular composition are different. How these differences affect the metabolic response, and in particular the lipid composition, in animals fed with these oils has not been clarified. The lipidomic analyses presented herein show similar changes in the hepatic content of the main lipid classes between the two oils and in both intervention groups an accumulation of hepatic TAGs was observed, with an accompanying decrease in the relative, but not absolute, levels of several PL classes. This confirms the observations reported in the previous paper from this study [13] where small, but not significant differences were observed in plasma and hepatic lipids between mice fed either FO or KO. The data presented here extends these findings with a more detailed analysis of the fatty acid composition and showing in particular some noteworthy differences in the minor lipids.

The changes in the hepatic composition of the main fatty acids were grossly a reflection of the higher content of omega-3 fatty acids in the oil containing feeds compared to the control feed. The abundance of 20:5 and 22:6 were increased in both intervention groups, but more in the FO than the KO group. This is likely a reflection of the respective feed compositions (lower omega-3 FA content in the KO diet), but the difference in abundance of these fatty acids is smaller than expected since the difference in the omega-3 content between these feeds are prominent [Table 1]. The relative increase was highest for 20:5, which is expected considering the very low hepatic level in the control mice and high content in the oils. The increased abundance of the omega-3 fatty acids was at the expense of other polyunsaturated fatty acids, with the decrease being particularly noteworthy for 18:2 and 20:4. In accordance with the highest increase in the relative content of omega-3 fatty acids in the FO mice, the decrease in 18:2 in PL and ether phospholipids (PC O, PC P, PE O) was larger in this group compared to the KO group. In contrast, the decreased abundance of 20:4 was largest in the KO fed mice (Fig. 2c). Arachidonic acid metabolites are key inflammatory intermediates and the lower level of this fatty acid in the KO fed mice indicate that KO may have a greater anti-inflammatory action than FO [16].

The observed increase in TAG species was dependent on their fatty acid composition, and was particularly large (up to15 fold) for TAG species containing the longer polyunsaturated FAs, particularly EPA and DHA (Fig. 2). For these species FO tended to result in a larger increase than KO. For other TAG species containing mono and di-unsaturated fatty acids and saturated FAs the increase in concentration was much smaller but still with the highest increase in the FO fed mice (Fig. 3a). This is consistent with the smaller decrease in 18:1 and 18:2 in the KO fed mice and a higher lipogenesis in the FO fed compared to the KO fed mice. Indeed, data that have been published from this study [13] showed a decreased lipogenesis and lower plasma FFAs level in the KO fed mice compared to the FO fed mice.

Differences between the intervention groups were particularly evident in some of the minor lipid classes, such as diacylglycrols, sphingolipids and plasmalogens, molecules that are associated with e.g., inflammation and insulin resistance. Sphingolipids have emerged as bioactive lipids with complex cellular functions, including roles in cell signaling as well as important structural roles in cellular membranes. Many sphingolipids have been shown to regulate cell growth, adhesion, migration, inflammation and intracellular trafficking [17, 18]. Cer is one of the component lipids making up sphingomyelin that play important roles as intracellular signaling molecules. Several lines of data indicate that the development of insulin resistance is associated with tissue accumulation of specific Cer species as well as accumulation of DAG [19–24]. DAGs are metabolites that have intracellular signaling properties, and hepatic insulin resistance have been reported to be associated with an increase in hepatic DAG content. The link between hepatic DAG accumulation and hepatic insulin resistance could be attributed to activation of PKCε, the predominant PKC isoform activated in liver following fat feeding. This mechanism is similar to the action of ceramides, which directly activate the PKCζ isoform that phosphorylates and inhibits the translocation of Akt/PKB, and thereby inhibits insulin signal transduction [19, 24, 25]. In our study the ceramides, especially GlcCer, were decreased significantly more in the krill oil compared to the fish oil group (Fig. 1). A similar trend was observed also for the hepatic DAG content. The abundance of DAG was decreased in the KO group and unchanged in the FO group (Fig. 1). Decreased accumulation of hepatic DAG and ceramides may be related to a reduced flux of especially saturated fatty acids. Cer synthesis is dependent on the availability of long-chain saturated FAs and excessive levels of free fatty acids stimulate the accumulation of Cer and various Cer metabolites, such as GlucCer and LacCer [20]. As reported previously, lipogenesis and plasma FFA levels seem to be decreased in the KO fed mice compared to the FO fed mice [13], which both could contribute to decreased synthesis of DAG, Cer and GlcCer. The decreased abundance of DAG and GlcCer in the KO fed compared to the FO fed mice may indicate that KO has a greater anti-inflammatory potential and a more beneficial effect on insulin sensitivity than FO.

Ceramides are synthesized by six different ceramide synthases (CerS) that differ in substrate specificity and tissue distribution. In this study most Cer were increased up to 2-fold in the brain but generally decreased in liver (Fig. 6), which may have physiological implications. The ceramide synthase 2 (CerS2) catalyzes synthesis of long-chain (22:0, 24:0, 24:1) Cer, and reduction in these ceramides is associated with neurodegenerative disorders including epilepsy [26]. A decrease in CerS2 activity in liver leads to compensatory increases in long-chain C16-ceramides that confers susceptibility to diet-induced steatohepatitis and insulin resistance [27]. C16-ceramides also promote weight gain and glucose intolerance [28]. However, there was a general trend towards decrease in all Cer species in liver. On the other hand, increased C16-ceramides in hypothalamus may promote lipotoxicity and ER-stress leading to sympathetic inhibition, reduced brown adipose tissue thermogenesis and weight gain [29].

Regarding LacCer the picture was slightly different. It is believed that a number of pro-inflammatory factors activate lactosylceramide synthase to generate LacCer, which in turn affect inflammatory states and processes such as adhesion and angiogenesis [30]. The FO fed mice showed an increased content of LacCer, indicating that FO, in contrast to KO, may induce pro-inflammatory factors even if the synthesis of anti-inflammatory prostaglandins are increased by omega-3 fatty acids.

Increased lipid oxidation accompanies pathological states such as type II diabetes and cardiovascular diseases that is associated with decreased plasmalogen levels. Plasmalogens contains a vinyl–ether bond that makes plasmalogens susceptible to oxidative attack. They may therefore have a crucial role as endogenous antioxidants during states of increased oxidative burden, especially in relation to membrane properties as these lipids are structural components of cellular membranes [31–33]. The hepatic level of the plasmalogen PC P was decreased by nearly 50 % in FO fed, but not changed in KO fed mice (Fig. 1b). The lower levels of plasmalogens in the FO mice may indicate a larger increase in oxidative stress compared to the KO mice.

Also for the hepatic and brain levels of CE differences between the intervention groups were observed. The hepatic levels of CE are determined by the cholesterol esterification reaction and secretion. Individual FAs interact with cholesterol to regulate both the output and uptake of sterol by the liver, and these effects are dependent on the esterification reaction [34]. This reaction is enhanced by unsaturated FA such as 18:1 and 18:2, and the hepatic abundance of these fatty acids is higher in the KO fed compared to the FO fed mice, consistent with the higher level of total CE in the KO mice (Figs. 2c and 6). KO and FO were similarly potent in regulation of CE 20:5 and 22:6 in brain and liver. The increase in CE 20:5 concentration in the liver was nearly 10 fold while the increase in CE 22:6 was much smaller. In the brain the specificity was opposite with the largest increase in CE 22:6. FO decreased CE 18:1 in the brain but not in the liver, while CE 20:4 was decreased in both tissues (Fig. 6). Two acyl-coenzyme A:cholesterol acyltransferases (ACAT1 and ACAT2) with different tissue expression catalyze the esterification of cholesterol to fatty acids [35]. ACAT2 expression is restricted to hepatocytes and enterocytes while ACAT1 is widely expressed. The different regulation of CE in liver and brain may be due to different fatty acid specificities of the two ACAT enzymes. DHA is substrate for ACAT1, which also inhibits CE formation from 18:1 (in line with the decrease in CE 18:1 in brain) [36]. However, contradictory to our observation that total CE is increased in KO (and slightly in FO), it was reported that both EPA and DHA decreased synthesis and secretion of CE in rat hepatocytes [37]. Taken together, the sum effect of KO and FO is likely to be due to changes in synthesis (substrate specificity/ inhibition), secretion and possibly also differences in transcriptional regulation of ACAT1 and ACAT2 by long chain PUFAs [38].

## Conclusion

This study has demonstrated that the molecular structure of omega-3 fatty acid esters may affect the distribution and fatty acid content of some of the major, and importantly also some of the minor, hepatic and brain lipids. Changes in the plasma lipid levels do not seem to be dependent on the source of the omega-3 fatty acids. However, considering the observed changes in the hepatic sphingolipid levels and fatty acid composition, especially the level of 20:4, the direction of change is always larger in the KO fed compared to the FO fed mice. The data are consistent with the hypothesis that KO has a stronger anti-inflammatory action and enhances insulin sensitivity more potently than FO.

## Methods

### Animals and diets

Nine to ten week old male C57BL/6 J mice were fed either a high-fat diet (HF) containing 24 % (wt/wt) fat (21.3 % lard and 2.3 % soy oil, n = 9), HF diet supplemented with FO (EPAX 6000 TG®, a generous gift of Epax A/S, Ålesund, Norway) (15.7 % lard, 2.3 % soy oil and 5.8 % FO, n = 6) or the HF diet supplemented with KO (Superba™, a generous gift of Aker BioMarine Antarctic AS, Oslo, Norway) (15.7 % lard, 2.3 % soy oil and 5.7 % KO, n = 6) and water ad libitum for 6 weeks. Diets were packaged in airtight bags and freeze stored until use to prevent lipid oxidation. Mice were housed in groups of three per cage at a constant temperature of 22 ± 2 °C and a light/dark cycle of 12/12 h. Body weights of the animals were measured approximately every seventh day and food intake was measured three times in the beginning of the 6-week study to optimize the food supply. At the end of study animals were fasted overnight, anesthetized with 2 % isoflurane (Schering-Plough, UK) and blood was collected by heart puncture. After collection all tissue samples were immediately frozen in liquid nitrogen and stored at −80 °C until further analysis. The animal experiments were carried out with ethical permission obtained from the Norway State Board for Biological Experiments and followed the Norwegian Research Councils ethical guidelines.

### Fatty acid analysis of diet composition

Lipids were extracted and methyl esters were obtained as previously described [39, 40]. After extraction into an organic solvent, fatty acid methyl esters were analyzed by gas–liquid chromatography. The gas chromatograph (GC 8000 TOP Finnigan, USA) was equipped with a programmed temperature vaporization injector, flame-ionization detector, AS 800 autosampler, and a fused silica capillary column coated with dimethylpolysiloxane stationary phase, DB1-ms (J & W Scientific, USA). Hydrogen was used as carrier gas. Column temperature was programmed from 110 to 310 °C with a gradient 2.5 °C/min. GC signal was acquired and evaluated with Chromeleon software (Dionex, USA). Peaks were identified by means of known FA standards and by means of mass spectra, obtained by GC/MS analysis (GCQ, Finnigan, USA) on the same column. Internal standard C21:0 was used for quantitation after calibration with known mixtures of FA standards.

### Lipidomic analysis

The tissue samples were weighted, pulverized with CP02 CryoPrep Dry Pulverization System (Covaris), and resuspended in ice-cold methanol containing 0.1 % butyl-hydroxytoluene (BHT) at a concentration of 100 mg/ml. The homogenized samples were stored at −80 °C prior to lipid extraction and analysis. Lipids were extracted from liver and brain homogenates using a modified Folch lipid extraction procedure [41] and in shotgun and TAG lipidomics lipid extracts were analyzed on a hybrid triple quadrupole/linear ion trap mass spectrometer (QTRAP 5500) equipped with a robotic nanoflow ion source (NanoMate HD) according to Ståhlman et al. [42]. Molecular lipids were analyzed in both positive and negative ion modes using multiple precursor ion scanning (MPIS) based methods [43, 44]. Targeted sphingolipid lipidomics was performed on a hybrid triple quadrupole/linear ion trap mass spectrometer (4000 QTRAP) equipped with an ultra high pressure liquid chromatography (UHPLC) system (CTC HTC PAL autosampler and Rheos Allegro pump) using multiple reaction monitoring (MRM) –based method [45].

### Statistical analyses

Statistical analyses were performed using SAS version 9.4. Log-transformation was applied to approximate log-normality of the data and unpaired Student *t*-test was performed to compare the differences between the study groups. Percentage differences and their significances were calculated with pairwise comparisons. Presented differences are the relative differences between group averages in non-log scale. In the statistical analyses p < 0.01 was considered significantly different.

## Abbreviations

EPA: Eicosapentaenoic acid
DHA: Docosahexaenoic acid
FA: Fatty acid
FO: Fish oil
KO: Krill oil
MUFA: Monounsaturated fatty acid
NEFA: Non-esterified fatty acid
PL: Phospholipid
PUFA: Polyunsaturated fatty acid
TAG: Triacylglycerol
CE: Cholesteryl ester
PC: Phosphatidylcholine
LPC: Lysophosphatidylcholine
LPL: Lysophospholipid
PC O: Ether-linked phosphatidylcholine
PE O: Ether-linked phosphatidylethanolamine
PC P: Plasmalogen phosphatidylcholine
PE P: Plasmalogen phosphatidylethanolamine
PS: phosphatidylserine
PE: Phosphatidylethanolamine
PG: Phosphatidylglycerol
PI: Phosphatidylinositiol
SM: Sphingomyelin
DAG: Diacylglycerol
Cer: Ceramide
LacCer: Lactosylceramide
Gal/GlcCer: Galactosyl- and glucosylceramide
Gb3Cer: Globotriaosylceramide
